# A General Chemical Ligation Approach Towards Isopeptide-Linked Ubiquitin and Ubiquitin-Like Assay Reagents

**DOI:** 10.1002/cbic.201100706

**Published:** 2011-12-23

**Authors:** Paul P Geurink, El Farid Oualid, Anika Jonker, Dharjath S Hameed, Huib Ovaa

**Affiliations:** Division of Cell Biology, Netherlands Cancer InstitutePlesmanlaan 121, 1066 CX Amsterdam (The Netherlands)

**Keywords:** activity assays, chemical ligation, fluorescent probes, proteases, protein–protein interactions

Ubiquitin (Ub) and ubiquitin-like proteins (Ubls) form a family of small and highly conserved post-translational modifiers that become linked to target proteins and thus modulate their function (such as degradation, trafficking and signalling).^1^ The linkage between a Ub(l) and a target protein most frequently consists of an isopeptide bond between the C-terminal carboxylate of Ub(l) and the ε-amine of a lysine residue. Ub(l) ligation requires the concerted action of enzymes E1, E2 and E3, defined combinations of which provide specificity for the protein target.^2^ Next to human Ub, 17 Ubls from nine phylogenetic classes have been reported.^3^ Each has its own discrete conjugation and deconjugation enzymes and has a distinct effect on its cellular target. The best-studied Ubls are Nedd8 and SUMO. For example, neddylation of cullin–RING E3 ligases is required for their enzymatic activity.^4^ The three human SUMO proteins (SUMO-1, SUMO-2 and SUMO-3) are conjugated to diverse target proteins, thereby often altering their interaction with other proteins through interactions between SUMO and SUMO-binding motifs.^5^

Specific deconjugating enzymes remove Ub and Ubls from target proteins. By doing so, they achieve three major functions.^6^ First, as Ub and Ubls are often translated as pro-proteins, they cleave the C termini of Ub and Ubls to generate the mature forms. Secondly, these proteases can reverse Ub(l) signalling functions and recycle free Ub and Ubls. Thirdly, in those cases where chains exist, such as for Ub and SUMO-2 and -3, proteases can perform a chain-editing function. As deregulation of Ub(l) deconjugating activity is linked to the occurrence of a variety of diseases, these are of interest as potential drug targets,^7^ and consequently, good assay reagents are required to report enzymatic activity and inhibition. Current assay reagents are mainly based on a Ub(l) part connected by a linear peptide bond to a reporter module—either a fluorogenic or latent enzyme that becomes active upon Ub(l) processing.7c In addition, besides lacking the native isopeptide linkage, such reagents cannot be functionalised (beyond the reporter module) to resemble a more physiologically relevant substrate.

A previously reported fluorescence anisotropy/fluorescence polarisation (FP) assay reagent for Ub(l) deconjugating enzymes is based on a fluorophore-labelled lysine, or a peptide linked to Ub by an isopeptide bond (Figure 1).^8^ This reagent has two characteristics that make it well-suited for high-throughput investigations of catalytic action.^9^ First, it is the only reported assay reagent that incorporates an isopeptide linkage;^8^ secondly, its physiological relevance (and potentially its affinity for a deconjugating enzyme) can be enhanced by functionalising the assay reagent with substrate-derived elements around the isopeptide linkage.^10^

**Figure 1:**
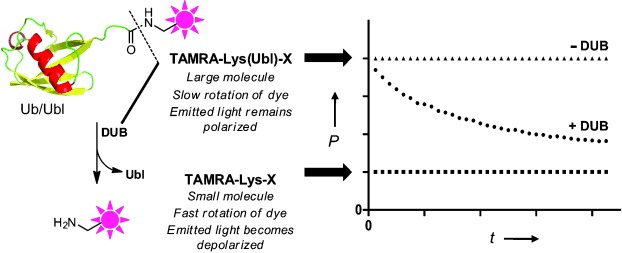
FP assay. When a fluorophore, covalently attached to a small molecule (e.g. a small peptide) is excited by polarised light, it will emit predominantly depolarised light. When it is bound to a high molecular weight molecule (e.g. Ub or a Ubl) the emitted light is much less depolarised. By following the change in fluorescence polarisation, the activity can be monitored. P, polarisation.

Because of the cumbersome enzymatic preparation required for this type of reagent, it has not become the standard in this field. To overcome the limitations set by enzymatic reactions, we and others recently reported methods for the site- and chemoselective Ub modification of peptides.^11^ In this approach, isopeptide-linked Ub-conjugates are prepared by native chemical ligation between a 5- or 4-thiolysine-containing peptide (**1**, Figure 2 B) and a Ub thioester. Desulfurisation of the intermediate thiolysine side-chain then affords the product with a native isopeptide linkage. The Ub E1 enzyme can be used to generate the required Ub thioester in situ.11c, 12 As E1 enzymes for most Ubls are commercially available, we wondered if the same strategy could also be used for the construction of Ubl-based conjugates. We started investigating the conjugation of the Ubl Nedd8 to a series of ten 5-thiolysine-containing peptides by using this method. The corresponding Nedd8–peptide conjugates were formed rapidly, with full conversion, as judged by SDS-PAGE analysis of the crude ligation mixtures (Figure 2 A). Treatment of the peptides with four other Ubls (SUMO-1, -2, -3 and ISG15) and their E1 enzymes under the same ligation conditions gave similar results (Figure S2 in the Supporting Information). Next, we tested whether our E1-mediated Ubl ligation could be used for the practical synthesis of various isopeptide-linked Ub(l)-based FP assay reagents.

**Figure 2:**
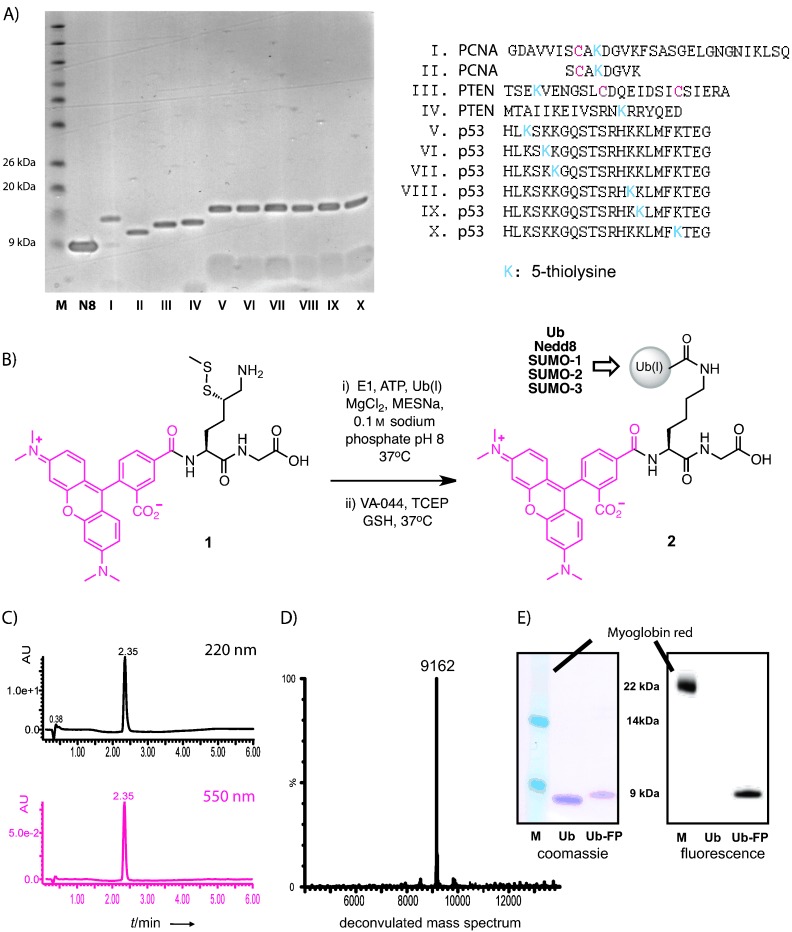
Ligations of Ub(l) with 5-thiolysine-modified peptides by E1-mediated Ub(l) ligation. A) Gel analysis of the crude ligation reactions in which Nedd8 (N8) was ligated to ten different peptides derived from PCNA, PTEN and p53. B) Construction of the Ubl-based FP reagents 2 from TAMRA-labelled dipeptide 1. C) HPLC, D) MS, and E) gel analysis of FP reagent 2-Ub after purification.

We started with the synthesis of Ub and Ubl (Nedd8, SUMO-1, SUMO-2 and SUMO-3) conjugates, which were natively linked through an isopeptide lysine bond to 5-carboxytetramethylrhodamine (TAMRA)-labelled 5-thioLys-Gly dipeptide (**2**, Figure 2 B). TAMRA-conjugate **1** (1 mm) and Ub(l) proteins (100 μm) were incubated with the appropriate E1 enzyme (150 nm) at 37 °C. In general, LC-MS and SDS-PAGE analysis showed full consumption of Ub(l) protein and formation of the desired ligation product after six hours. Next, the crude ligation product was desulfurised by addition of the radical initiator VA-044 (20 mm), tris-(2-carboxyethyl)phosphine (TCEP, 150 mm) and glutathione (40 mm) with overnight incubation at 37 °C.^13^ After HPLC purification and lyophilisation, the desired products were obtained in overall yields of 20–50 %. All purified products were analysed by LC-MS and SDS-PAGE (see Figure 2 C and the Supporting Information). As all three SUMO proteins contain a native cysteine residue, we anticipated that this might be desulfurised to an alanine residue. However, in all cases LC-MS analysis showed desulfurisation of the thiolysine moiety only, even after prolonged treatment with the desulfurisation cocktail.11a, b, 14

Next, we tested the FP reagents in deconjugation assays by treating them with three human deubiquitylating enzymes (DUBs; UCH-L3, USP7/HAUSP and USP21), two viral ovarian tumour domain (OTU) DUBs and three SUMO-specific proteases (SENP1, SENP6 and SENP7). These proteases were incubated at six different concentrations with all five Ub(l) FP reagents (**2**, Figure 2 B) at room temperature (Figure 3 and the Supporting Information). The completely hydrolysed product (TAMRA-Lys-Gly) was used as a control, and the spectroscopic optics were calibrated by applying an FP value of 50 mP (millipolarisation units) for this tracer. As expected, the Ub FP reagent was efficiently cleaved by all tested deubiquitinases in a concentration-dependent manner, and was unaffected by all three SUMO-specific proteases. The most active DUB in this series appeared to be UCH-L3, which almost completely processed the Ub FP reagent within 80 min at 15.6 pm (Figure 3 A). The *K*_m_ and *k*_cat_ values were determined by measuring fluorescence polarisation over time at different substrate concentrations (Figure 3 B, Table 1). Michaelis–Menten plots revealed *k*_cat_ and *K*_m_ values that are comparable with those reported for the fluorogenic ubiquitinamidomethyl coumarin Ub-AMC (*k*_cat_=9.1 s^−1^, *K*_m_=51 nm).^15^

**Figure 3:**
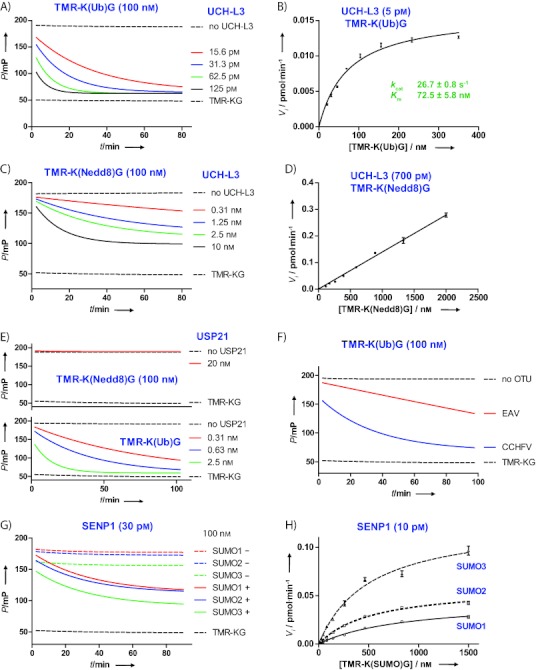
FP assays with Ub(l) FP reagents and different Ub(l) deconjugating enzymes. A) Reaction time-course and B) substrate titration for UCH-L3 and the Ub-derived FP reagent. C) Reaction time course and D) substrate titration for UCH-L3 and the Nedd8-derived FP reagent. E) Reaction time course for USP21 and the Nedd8- (top) and Ub-derived (bottom) FP reagents. F) Reaction time course for the EAV- and CCHFV-derived OTUs and the Ub-derived FP reagent. G) Reaction time course for the SUMO1- ,2- and 3-derived FP reagents in the presence (+) or absence (−) of SENP1. H) Substrate titration for SENP1 and the SUMO1-, 2- and 3-derived FP reagents. Additional results for all tested Ub(l) deconjugating enzymes with different Ub(l) FP reagents at different concentrations are presented in the Supporting Information. *P*: polarisation.

**Table 1:** Kinetic analysis of Ub(l) proteases for Ub(l) FP substrates.

Enzyme	FP substrate	*k*_cat_ [s^−1^]	*K*_m_ [nm]	*k*_cat_/*K*_m_ [m^−1^ s^−1^]
UCH-L3	Ub	27±0.8	73.0±5.8	3.7×10^8^
SENP1	SUMO-1	38±2.1	876±93	4.3×10^7^
SENP1	SUMO-2	50±1.7	540±41	9.2×10^7^
SENP1	SUMO-3	106±5.2	508±59	2.1×10^8^
USP7	Ub	3.3±0.22	34 600±3400	9.5×10^4^
USP7	Ub-PTEN[5–21]	5.0±0.21	22 900±1600	2.2×10^5^

UCH-L3 is known to exhibit deneddylating activity.^16^ The Nedd8-based FP reagent was indeed processed by UCH-L3, although with a lower efficiency than Ub (Figure 3 C and D); this is in line with an earlier report.^15^ It must be noted that the catalytic breakdown of the Nedd8-based FP reagent stopped at around 60 % conversion (Figure 3 C). This was also observed for other substrates (vide infra). Currently, the reason for this remains unclear. However, an activity assay using excess Ub showed that this effect cannot be explained by product inhibition (Figure S23). Loss of enzymatic activity over time also does not account for this, as the final mP was independent of enzyme concentration (Figure 3 A). Nonetheless, both the Ub and Nedd8 FP reagents are well suited for monitoring UCH-L3 activity. USP21 has also been reported to deconjugate both Ub and Nedd8,^17^ although a more recent report shows that USP21 exhibits no deneddylating activity.^18^ Our results show that USP21 processes the Ub FP reagent at sub-nanomolar concentrations (Figure 3 E, bottom) but not the Nedd8 FP reagent, even at 20 nm (Figure 3 E, top), which supports the recent report.

We investigated two viral deubiquitinases that belong to the OTU class, one from equine arteritis virus (EAV) and one from the Crimean–Congo haemorrhagic fever virus (CCHFV). CCHFV is a lethal human pathogen, and it is believed that its inherent DUB activity has a major role in its pathogenic function.^19^ Both OTU DUBs were found to efficiently cleave the Ub FP substrate in a concentration-dependent manner (Figure 3 F), but they lacked reactivity towards Nedd8^20^ and SUMO FP reagents (Figures S14 and S15).

As shown, all tested deubiquitinases efficiently processed the Ub FP reagent, however they proved to be unreactive towards the three SUMO-derived reagents. In contrast, the SUMO-specific protease, SENP1, was unreactive towards the Ub FP reagent but efficiently processed the three SUMO FP reagents at 30 pm and with comparable efficiency (Figures 3 G and S16), although there was a slight preference for SUMO-3 (Figure 3 H and Table 1). We also tested SENP6 and SENP7 for their ability to process the SUMO-derived FP reagents. It is known that SENP6 and SENP7 exhibit specificity for SUMO-2 and SUMO-3, whereas SENP1 lacks a clear preference for any particular SUMO isoform.^21^ Indeed, SENP6 and SENP7 properly processed both the SUMO-2 and SUMO-3 FP reagents, albeit with a clear preference for SUMO-3 (Figures S17 and S18) in both cases. The SUMO-1 FP reagent was not processed by SENP6 or SENP7 at up to 20 nm.

We further functionalised our FP reagents by introducing a peptide sequence derived from a known ubiquitylated substrate. These context-specific reagents resemble the native environment that a Ub(l) protease encounters better than a single lysine residue. As a test case, our attention was drawn to the tumour suppressor phosphatase PTEN, which contains two major monoubiquitylation sites (Lys13 and Lys289). Monoubiquitylation of these sites is important for regulation of PTEN-mediated tumour suppression and its nuclear import.^22^ The major DUB responsible for PTEN deubiquitylation is USP7/HAUSP.^23^ Based on the peptide sequences surrounding Lys13 and Lys289, we designed two FP reagents that comprised a TAMRA-labelled 17-amino-acid PTEN peptide (i.e., PTEN[5–-21] and PTEN[281-–297], respectively). The lysine residues were linked by an isopeptide bond to Ub. The ability of full-length USP7 to hydrolyse these FP reagents was assessed in an FP assay at different concentrations of USP7 (Figure 4 and Figure S20). For comparison, all other Ub(l)-derived reagents were also tested. As expected, USP7 could not process the Ubl-derived FP reagents (e.g., Nedd8 and SUMO, Figure S11) but efficiently hydrolysed the TAMRA-Lys(Ub)-Gly reagent. Whereas USP7 showed high activity against the PTEN[5–21]-based FP reagent, it was much less active on the PTEN[281–297]-based FP reagent (Figures 4 and S20). This result was also apparent from a gel-based assay (Figure S21), thereby confirming that the observed difference in reactivity depends on the nature of the FP substrate. It was previously reported that both Lys13 and Lys289 are deubiquitylated by USP7 in vivo.^23^ However, to the best of our knowledge, the relative USP7 deubiquitylation rates for these sites are not known. The observed differences in USP7 reactivity here might be explained by an intrinsic preference of USP7 for monoubiquitylated Lys13, although further experiments are needed to substantiate this. The kinetic data for the PTEN[5–21]-derived FP substrate revealed that introduction of the PTEN[5–21]-peptide resulted in a higher *k*_cat_/*K*_m_ value compared with the unfunctionalised TAMRA-Lys(Ub)-Gly reagent (Table 1 and Figure S22). The kinetic parameters for the PTEN[281–297] reagent were not determined as it was only minimally processed by USP7. It must be noted that introduction of the larger peptides around the isopeptide linkage decreases the dynamic range of the FP assay reported here. However, with a calculated *Z*-score of 0.88 for the PTEN[5–21]-based FP reagent, we believe that the system is well suited for monitoring Ub(l) proteolysis activities in a context-specific manner.^24^

**Figure 4:**
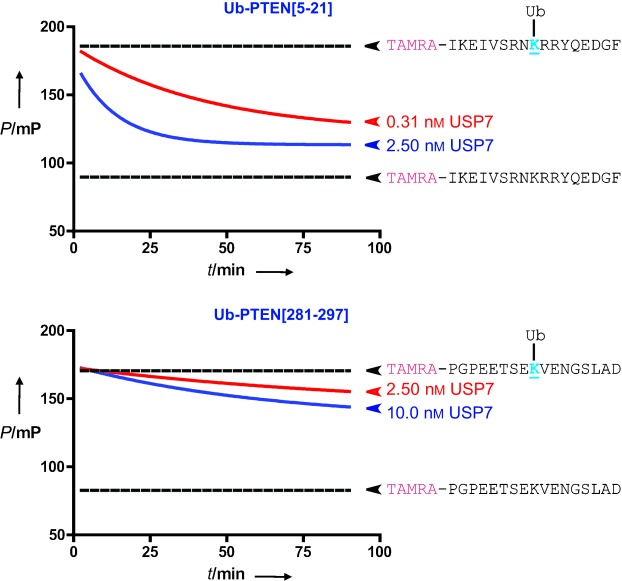
FP assays with full-length USP7 and ubiquitylated PTEN[5–21] (top) and PTEN[281–297] (bottom) derived peptide FP substrates (100 nm). P: polarisation.

In conclusion, we have shown that the 5-thiolysine-mediated ligation can be used to generate a wide range of isopeptide-linked Ub(l)-based FP assay reagents, something that was not possible with conventional enzyme-based strategies. We and others have demonstrated that these are very powerful tools for measuring Ub(l) deconjugating activity.^8^ In principle, our methodology can be adapted to any Ubl for which the E1 enzyme is available. In contrast to any other reagent, it is possible to incorporate substrate-based peptide sequences. This is a major advantage, as it offers the possibility of more physiologically relevant assay reagents.7c Overall, the practical generation of FP assay reagents described here will likely “catalyse” both fundamental research in the Ubl field and drug discovery efforts.

## Supporting information

Detailed facts of importance to specialist readers are published as ”Supporting Information”. Such documents are peer-reviewed, but not copy-edited or typeset. They are made available as submitted by the authors.

## References

[1] Hochstrasser M (2009). Nature.

[2] Behrends C, Harper JW (2011). Nat. Struct. Mol. Biol.

[3] Kerscher O, Felberbaum R, Hochstrasser M (2006). Annu. Rev. Cell Dev. Biol.

[4] Soucy TA, Smith PG, Rolfe M (2009). Clin. Cancer Res.

[5] Gareau JR, Lima CD (2010). Nat. Rev. Mol. Cell Biol.

[6] Reyes-Turcu FE, Wilkinson KD (2009). Chem. Rev.

[7a] Bedford L, Lowe J, Dick LR, Mayer RJ, Brownell JE (2011). Nat. Rev. Drug Discovery.

[7b] Goldenberg SJ, McDermott JL, Butt TR, Mattern MR, Nicholson B (2008). Biochem. Soc. Trans.

[7c] Shanmugham A, Ovaa H (2008). Curr. Opin. Drug Discov. Devel.

[8] Tirat A, Schilb A, Riou V, Leder L, Gerhartz B, Zimmermann J, Worpenberg S, Eidhoff U, Freuler F, Stettler T, Mayr L, Ottl J, Leuenberger B, Filipuzzi I (2005). Anal. Biochem.

[9] Huang X, Aulabaugh A (2009). Methods Mol. Biol.

[10] Shanmugham A, Fish A, Luna-Vargas MPA, Faesen AC, El Oualid F, Sixma TK, Ovaa H (2010). J. Am. Chem. Soc.

[11a] Kumar KSA, Haj-Yahya M, Olschewski D, Lashuel HA, Brik A Angew. Chem.

[11b] El Oualid F, Merkx R, Ekkebus R, Hameed DS, Smit JJ, de Jong A, Hilkmann H, Sixma TK, Ovaa H Angew. Chem.

[11c] Ovaa H, El Oualid F

[11d] Yang R, Pasunooti KK, Li F, Liu X-W, Liu C-F (2009). J. Am. Chem. Soc.

[12] Burchak ON, Jaquinod M, Cottin C, Mugherli L, Iwai K, Chatelain F, Balakirev MY (2006). ChemBioChem.

[13] Wan Q, Danishefsky SJ Angew. Chem.

[14] Kumar KSA, Spasser L, Erlich LA, Bavikar SN, Brik A Angew. Chem.

[15] Gan-Erdene T, Nagamalleswari K, Yin L, Wu K, Pan ZQ, Wilkinson KD (2003). J. Biol. Chem.

[16] Wada H, Kito K, Caskey LS, Yeh ETH, Kamitani T (1998). Biochem. Biophys. Res. Commun.

[17] Gong L, Kamitani T, Millas S, Yeh ETH (2000). J. Biol. Chem.

[18] Ye Y, Akutsu M, Reyes-Turcu F, Enchev RI, Wilkinson KD, Komander D (2011). EMBO Rep.

[19] Akutsu M, Ye Y, Virdee S, Chin JW, Komander D (2011). Proc. Natl. Acad. Sci. USA.

[20] Capodagli GC, McKercher MA, Baker EA, Masters EM, Brunzelle JS, Pegan SD (2011). J. Virol.

[21] Lima CD, Reverter D (2008). J. Biol. Chem.

[22] Trotman LC, Wang X, Alimonti A, Chen Z, Teruya-Feldstein J, Yang H, Pavletich NP, Carver BS, Cordon-Cardo C, Erdjument-Bromage H, Tempst P, Chi S-G, Kim H-J, Misteli T, Jiang X, Pandolfi PP (2007). Cell.

[23] Song MS, Salmena L, Carracedo A, Egia A, Lo-Coco F, Teruya-Feldstein J, Pandolfi PP (2008). Nature.

[24] Zhang JH, Chung TD, Oldenburg KR, The *Z*-score is a statistic factor used to quantify the quality of an assay. A good assay has a *Z*-score >0.5, with 1.0 being the maximum; see: (1999). J. Biomol. Screening.

