# Supplementary material for: A General Chemical Ligation Approach Towards Isopeptide-Linked Ubiquitin and Ubiquitin-Like Assay Reagents
Source: Chembiochem. 2011 Dec 23;13(2):293–7. doi: 10.1002/cbic.201100706 (PMC3488293; doi:10.1002/cbic.201100706)

CHEMBIOCHEM

Supporting Information

© Copyright Wiley-VCH Verlag GmbH & Co. KGaA, 69451 Weinheim, 2012

Ubiquitin-Based Probes Prepared by Total Synthesis To Profile the Activity of Deubiquitinating Enzymes

Annemieke de Jong,[a] Remco Merkx,[a] Ilana Berlin,[a] Boris Rodenko,[a]
Ruud H. M. Wijdeven,[a] Dris El Atmioui,[a] Zeliha Yalçin,[a] Craig N. Robson,[b]
Jacques J. Neefjes,[a] and Huib Ovaa*[a]

cbic_201200497_sm_miscellaneous_information.pdf

Supplementary Information

Figure S1. **Side by side comparison Ub synthesis using two or six dipeptide analogues and HCTU or PyBOP as coupling agent.** The $Ub_{(1\text{-}76)}$ peptide sequences were synthesized in parallel on 25 µmol scale starting with Fmoc-Gly Trityl resin. Fmoc deprotection was performed 3x using 20% piperidine in NMP (1x5 min., 1x10 min., 1x5 min.), followed by washing the resin 6x with NMP. Coupling of Fmoc-protected amino acids was achieved by adding a pre-activated mixture to the resin of 4 equiv. of Fmoc-protected amino acid, 4 equiv. of PyBOP or HCTU and 8 equiv. of DIPEA. After 45 min. the resin was washed 3x with NMP. Coupling of pseudoproline building blocks on positions Leu8-Thr9, Ile13-Thr14, Leu56-Ser57 and Ser65-Thr66, and dimethoxybenzyl (Dmb) dipeptides on positions Ala46-Gly47 and Asp52-Gly53, was extended to two hours. In the comperative syntheses using only two dipeptide analogues, a pseudoproline building block was used on position Leu56-Ser57 and a Dmb dipeptide was introduced on position Asp52-Gly53. After the last cycle, resin was washed 4x with diethyl ether (Et_2O) and dried at high vacuum. To cleave the Ub from the resin a mixture of TFA, phenol, triisopropylsilane (TIPS) and water (90:2.5:2.5:5) was added to the resin and shaken for three hours. The resin was filtered and the filtrate was precipitated in cold Et_2O:n-pentane (3:1). The precipitate was isolated by centrifugation for 6 min. at 1500 g at 4˚C and washed by 3 repetitive cycles of resuspension in ice-cold Et_2O and centrifugation. Finally, the pellet was taken up in water:acetonitrole:acetic acid (65:25:10), frozen and lyophilized. We analyzed the products by analytical HPLC (A), LC-MS (B, Left panels: MS analysis of Ub synthesized under various conditions. Right panels: Deconvoluted spectra; calculated $[M+H]^+$ 8560.6 Da, found $[M+H]^+$ 8558.0 Da/8559.0 Da.), and by SDS page followed by Coomassie Brilliant Blue staining (C).

We conclude that higher quality products of the linear Ub synthesis are obtained when using six dipeptide analogues as compared to the use of two of these building blocks as claimed in the paper of Bavikar et al.*

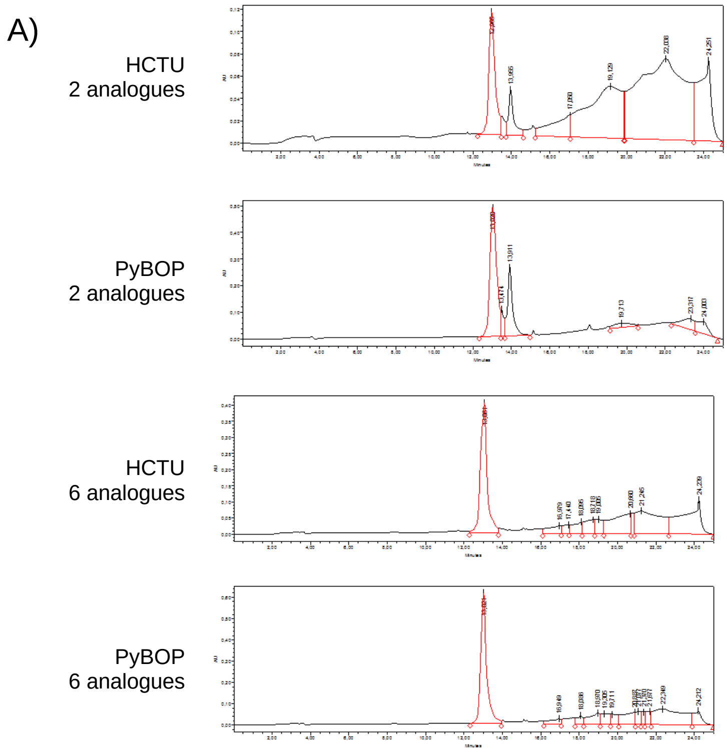

* S. N. Bavikar, L. Spasser, M. Haj-Yahya, S. V. Karthikeyan, T. Moyal, K. S. Kumar, A. Brik, *Angew Chem Int Ed Engl* 2012, *51*, 758-763.

B)

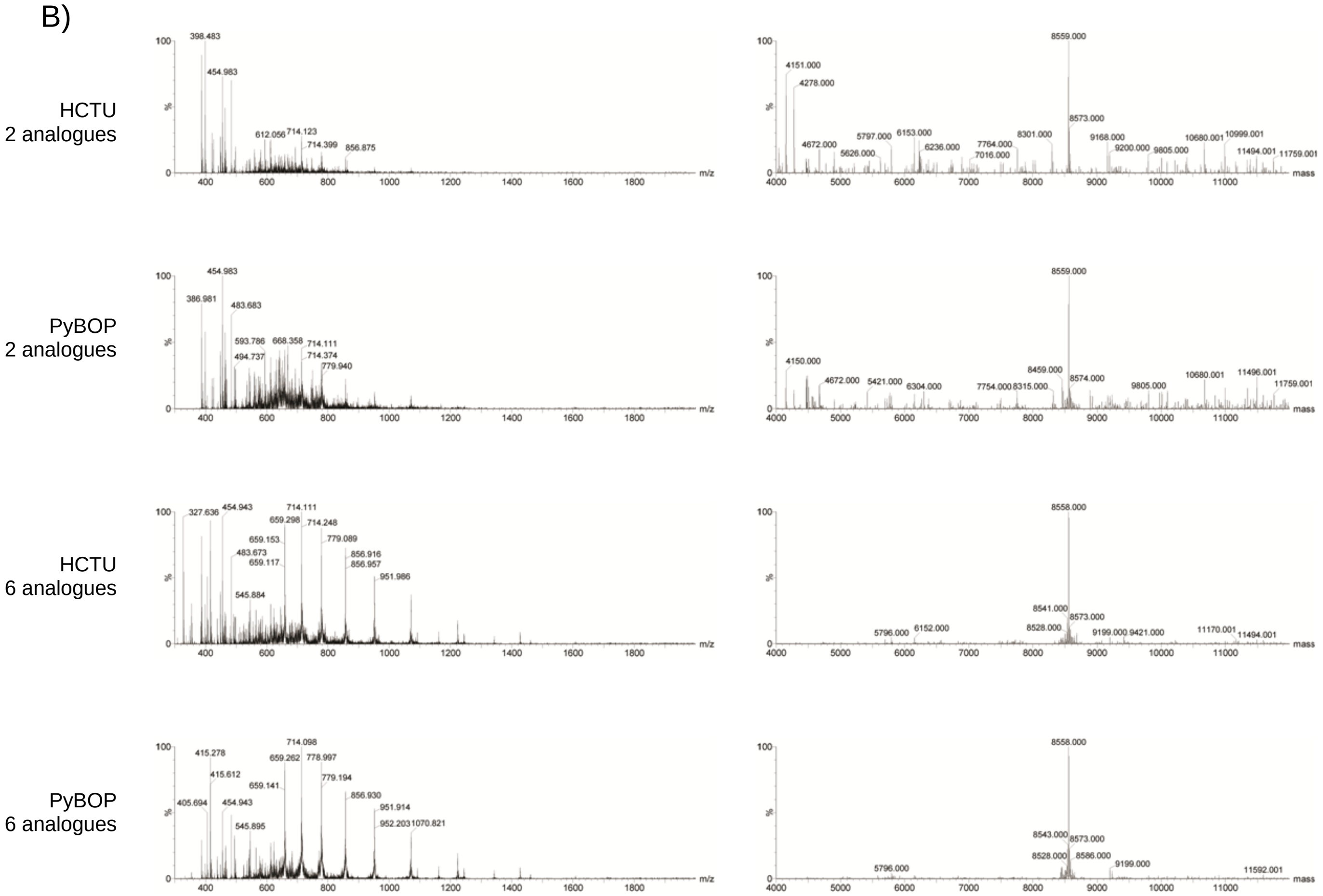
HCTU
2 analogues
PyBOP
2 analogues
HCTU
6 analogues
PyBOP
6 analogues
m/z
mass

C)

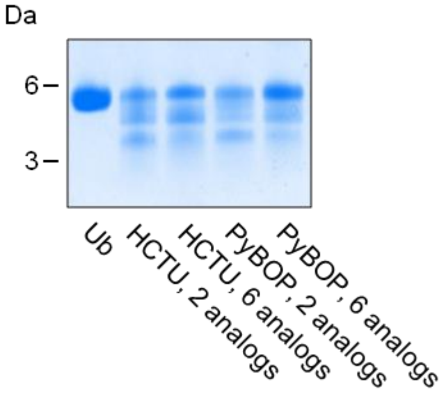
Da
6
3
Ub
HCTU, 2 analogs
HCTU, 6 analogs
PyBOP, 2 analogs
PyBOP, 6 analogs

Figure S2. **Structures of Fmoc-amino acid residues and tags that were incorporated in the chemically synthesized DUB probes.**

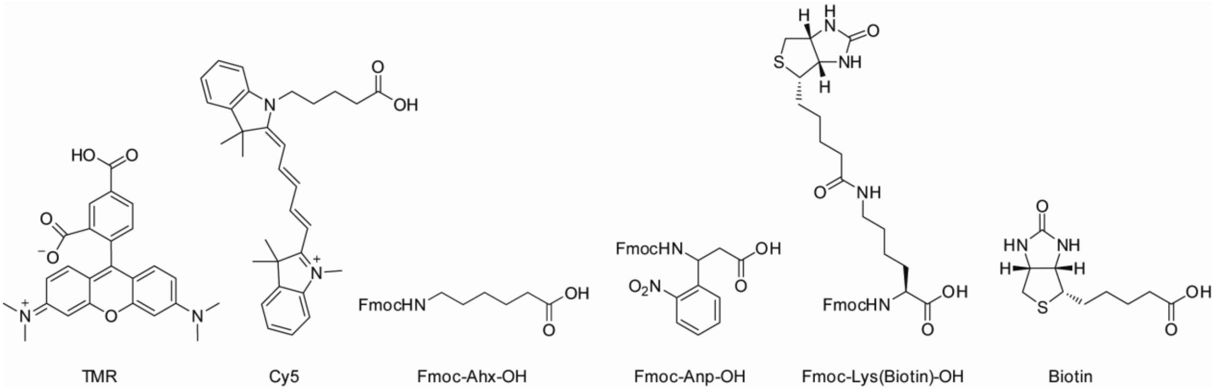

Figure S3. **Mass spectrometric characterization of HA-Ahx$_2$UbVME 1.** A) Liquid chromatography profile of purified probe **1**. B) MS analysis of purified probe **1**. MS analysis was carried out for the main peak only. C) Deconvoluted spectrum; calculated $[M+H]^+$ 9910.3 Da, found $[M+H]^+$ 9912.0 Da.

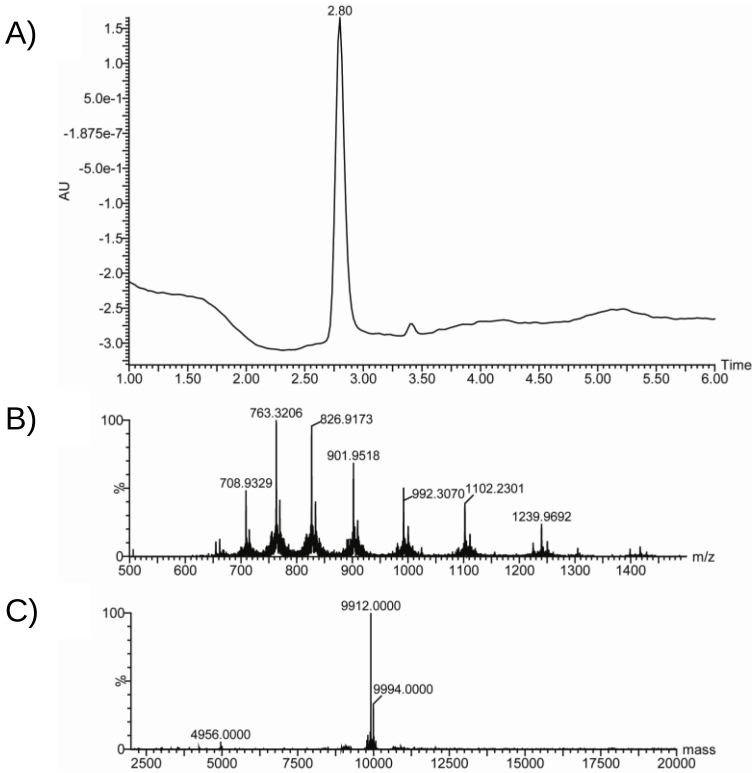

Figure S4. **Mass spectrometric characterization of TMRUbVME 2.** A) Liquid chromatography profile of purified probe **2**. B) MS analysis of purified probe **2**. MS analysis was carried out for the main peak only. C) Deconvoluted spectrum; calculated $[M+H]^+$ 9012.8 Da, found $[M+H]^+$ 9014.0 Da.

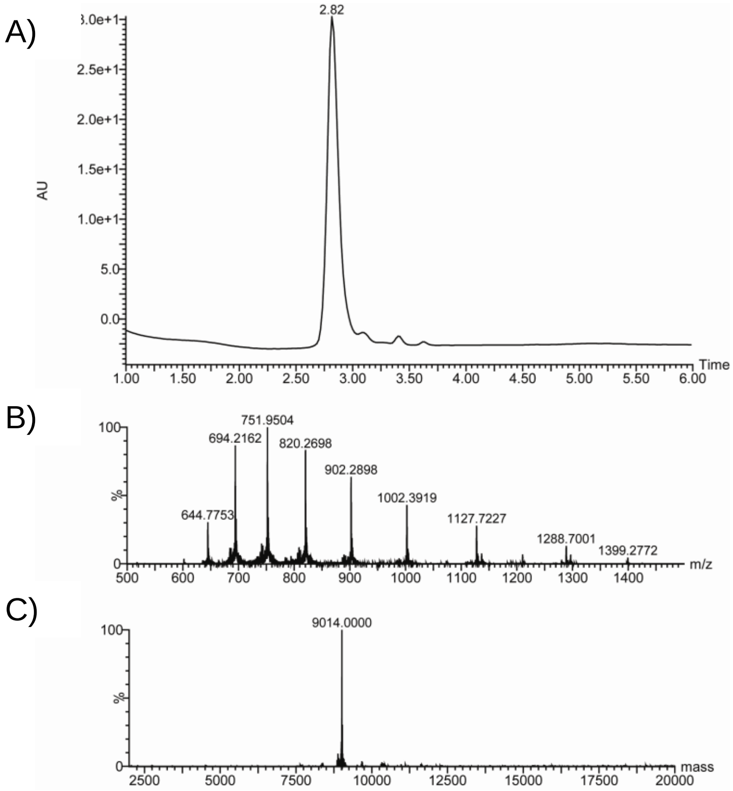

Figure S5. **Mass spectrometric characterization of Cy5AhxHis$_6$UbVME 3.** A) Liquid chromatography profile of purified probe **3**. B) MS analysis of purified probe **3**. MS analysis was carried out for the main peak only. C) Deconvoluted spectrum; calculated $[M+H]^+$ 9987.4 Da, found $[M+H]^+$ 9987.0 Da.

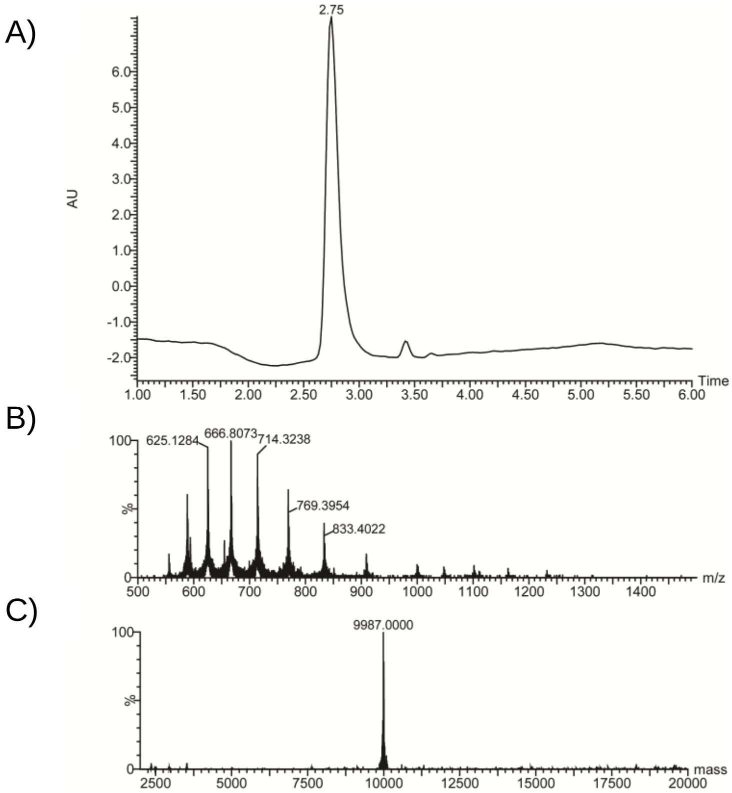

Figure S6. **Mass spectrometric characterization of Cy5Lys(Biotin)AhxUbVME 4.** A) Liquid chromatography profile of purified probe **4**. B) MS analysis of purified probe **4**. MS analysis was carried out for the main peak only. C) Deconvoluted spectrum; calculated $[M+H]^+$ 9519.2 Da, found $[M+H]^+$ 9519.0 Da.

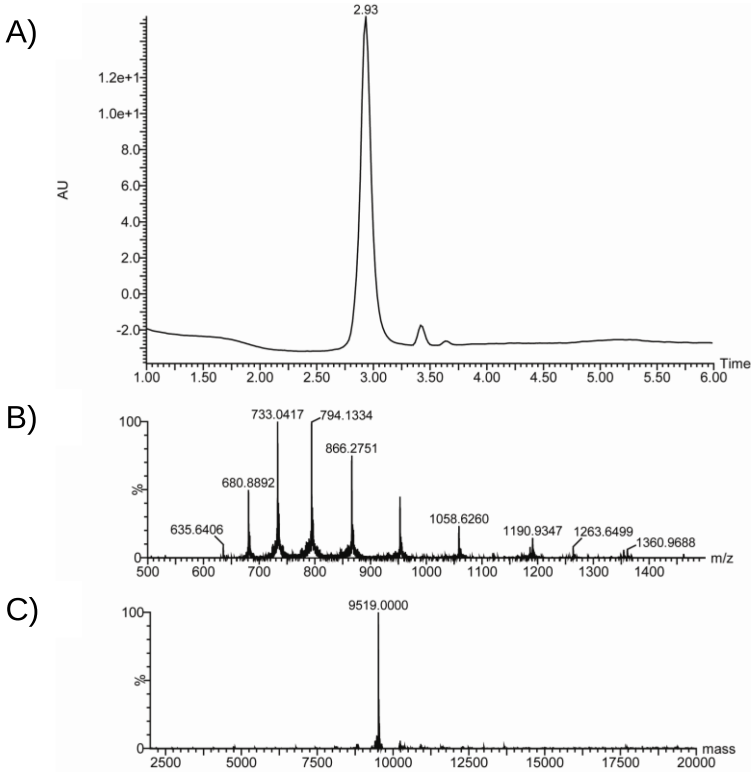

Figure S7. **Mass spectrometric characterization of BiotinAnpUbVME 5.** A) Liquid chromatography profile of purified probe **5**. B) MS analysis of purified probe **5**. MS analysis was carried out for the main peak only. C) Deconvoluted spectrum; calculated $[M+H]^+$ 9018.8 Da, found $[M+H]^+$ 9020.0 Da.

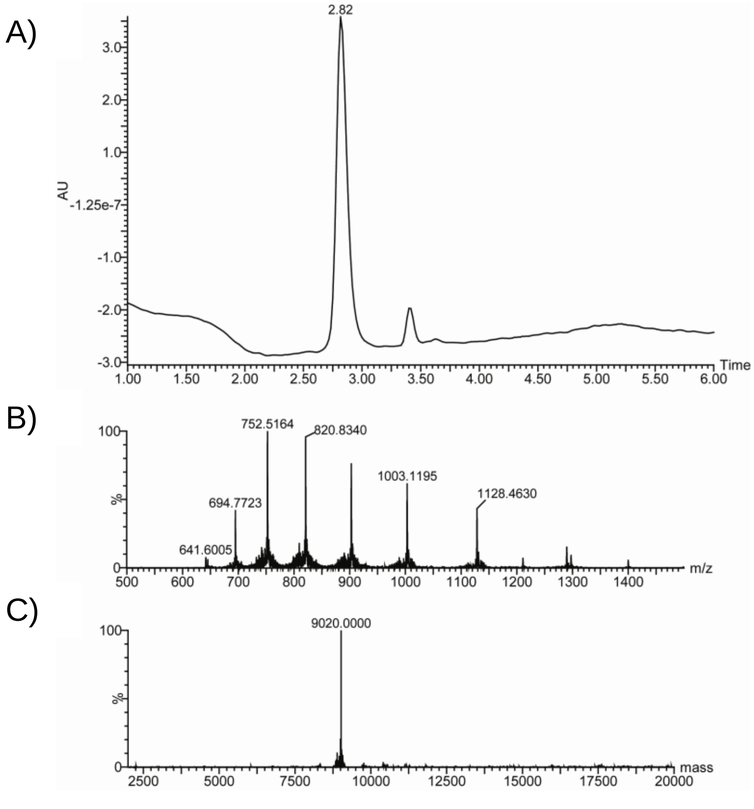

Figure S8. **Mass spectrometric analysis of impurities found in LC-MS spectra of UbVME probes (Figures S3-S7).** The additional peaks shown in the LC spectra of the UbVME probes originate from an impurity on the column, as this peak is also shown when a blank is run (lowest panel).

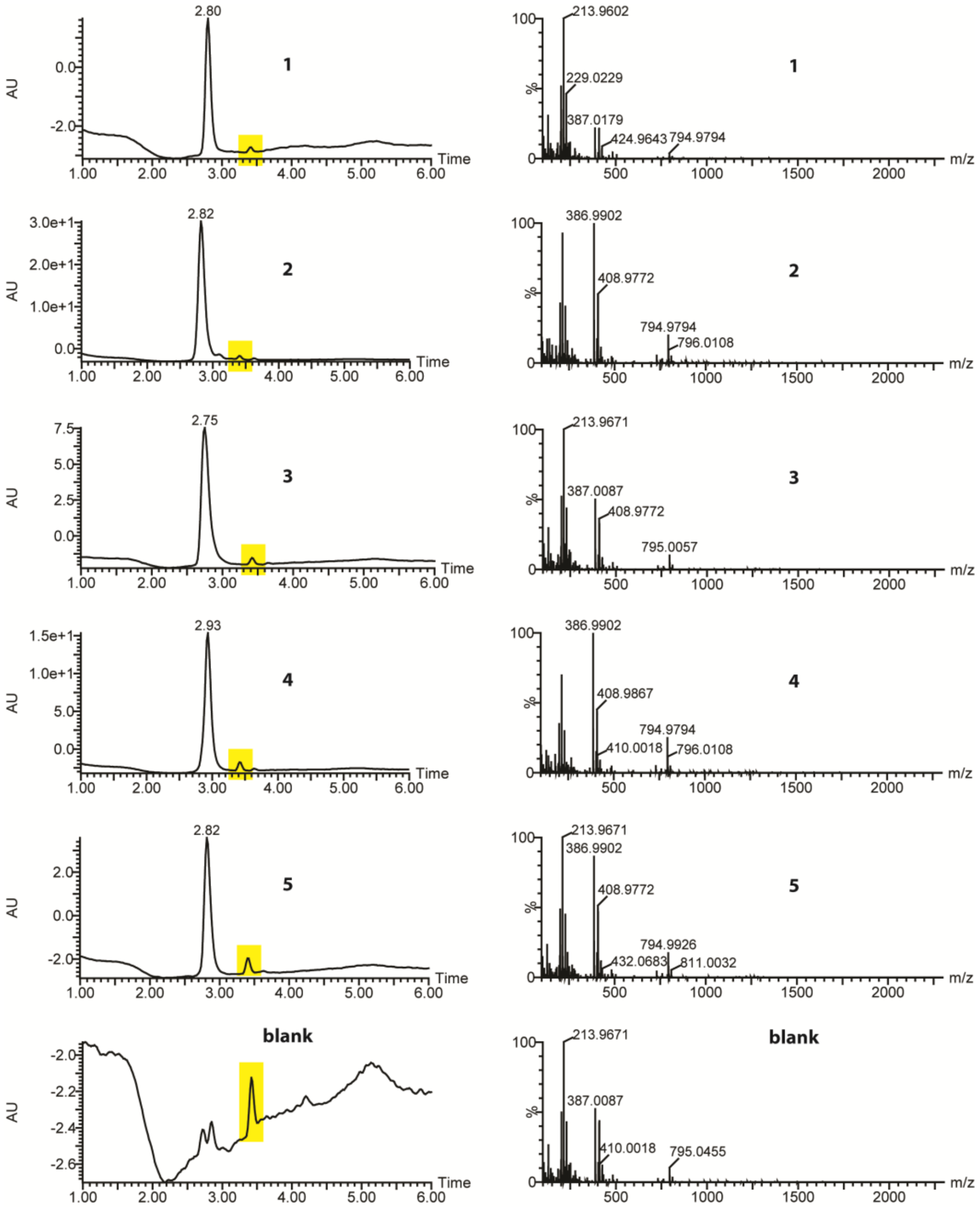

Figure S9. **Analytical HPLC profile of probe 3.** The analytical HPLC run of probe **3** does not show the impurity seen when the probe was analyzed by LC-MS (Figure S5 and S8).

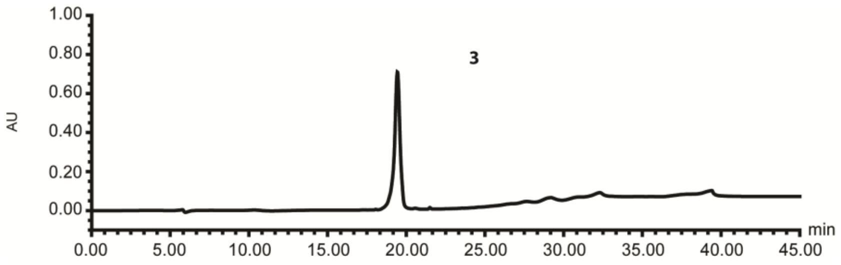

Figure S10. **DUB profiling in cell extract using both classically and chemically synthesized UbVME probes.** A) EL4 cell lysate incubated with first generation probe HAUbVME (HAUbVME (expr)), obtained by the conventional intein method, and with the chemically synthesized, HA-tagged, probe **1**. Proteins were separated by SDS-PAGE and analyzed by immunoblotting using primary antibodies rabbit anti-HA and mouse anti-β-actin in combination with fluorescent secondary antibodies anti-mouse 680 and anti-rabbit-800 and visualized by fluorescence scanning. Actin is probed as a loading control. The bands observed at 42 kDa orignate from leaking from the actin signal. B) EL4 lysate incubated with second generation probe **2**. Proteins were separated by SDS-PAGE and analyzed directly by in-gel fluorescence scanning.

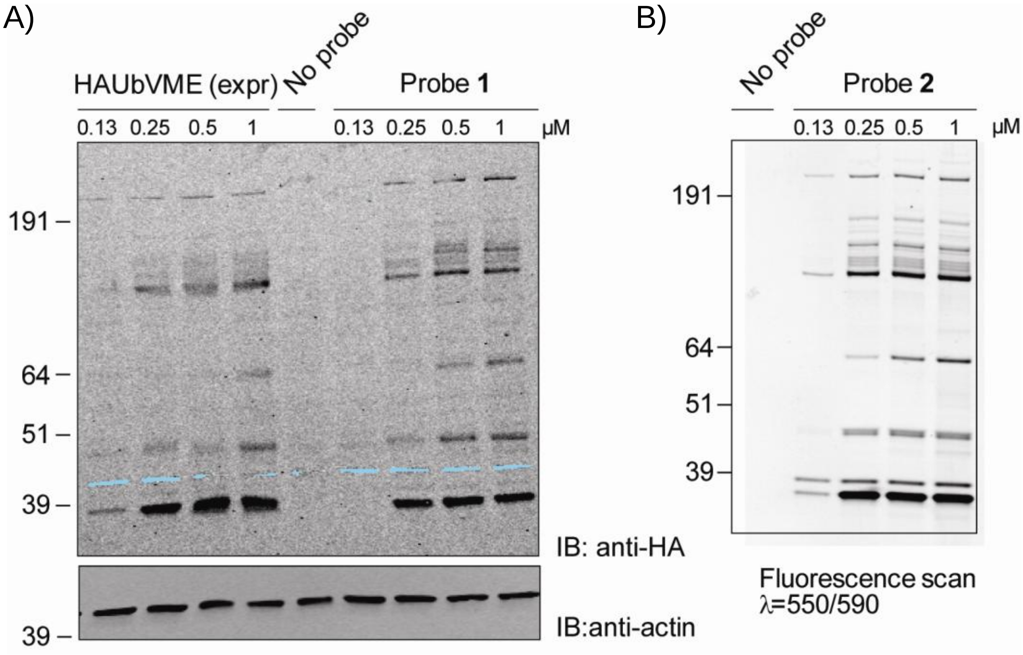

Figure S11. **Dual activity probe 4 containing both a fluorophore and a biotin tag can be used for detection of DUBs in cell lysates.** DUBs present in EL4 lysates were detected using indicated concentrations of the tandem-tagged probe **4**, either by in-gel fluorescent scanning (left panel) or by Western blotting followed by streptavidin-poly-HRP staining (right panel). Similar labeling patterns are observed.

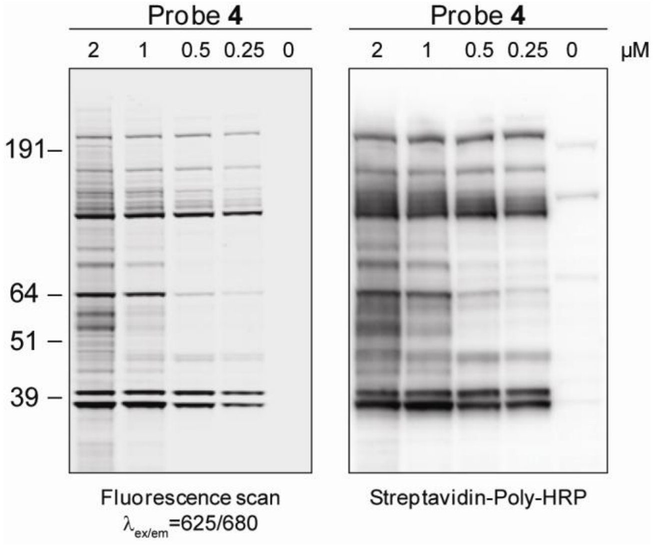

Figure S12. **Fluorescent DUB activity probe 2 allows for rapid profiling of DUB inhibitors**. EL4 lysates were incubated with indicated concentrations of small molecule DUB inhibitors compound A, WP1130, b-AP15 and PR-619 at 37˚C for 1.5 h. Subsequently, lysates were labeled with 0.5 µM probe **2** at ambient temperature for 15 min and remaining DUB activity was visualized by in-gel fluorescent scanning.

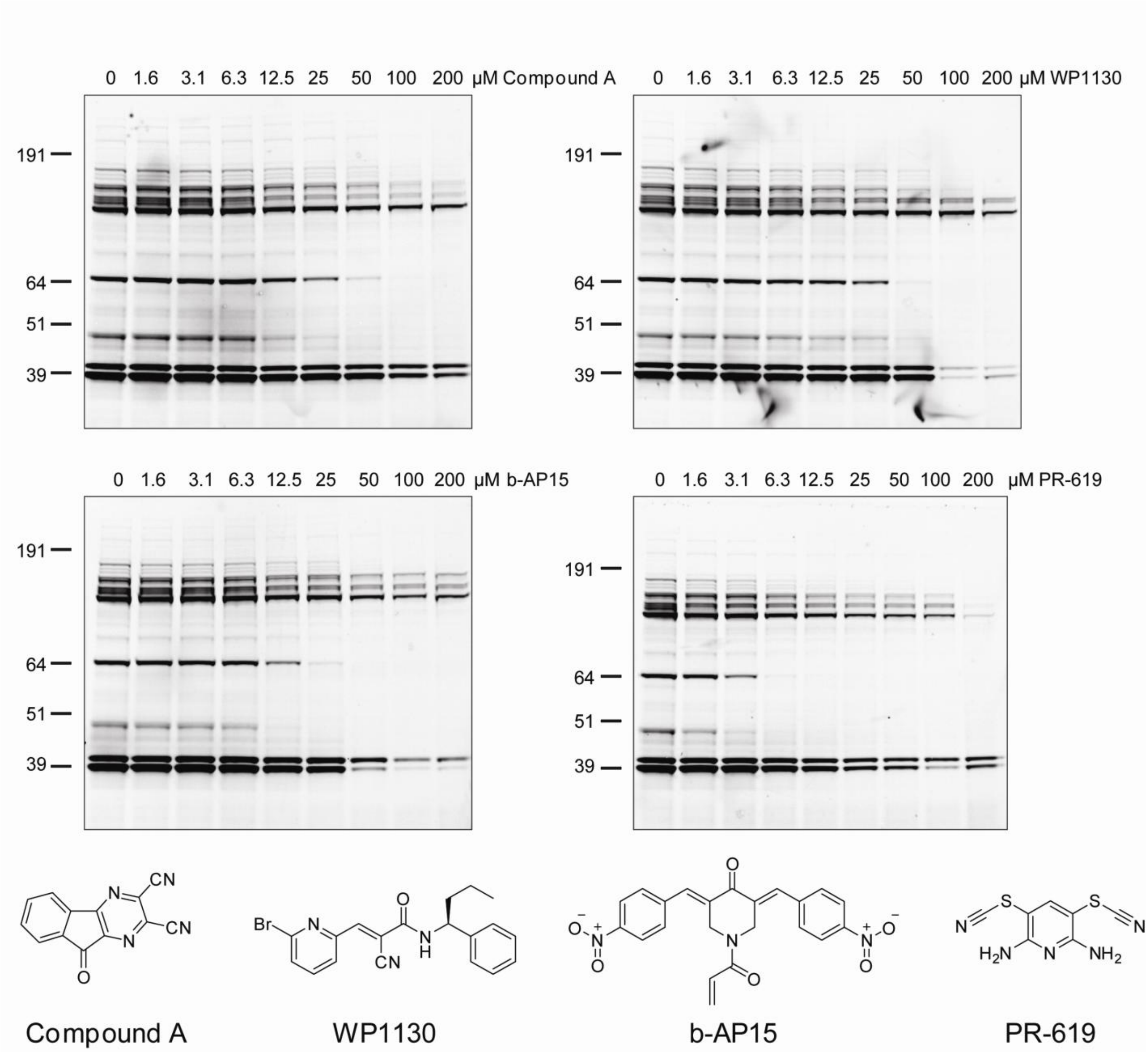

Figure S13. **Second generation probe allows for monitoring biological manipulations of specific DUBs.** MelJuSo cells were transfected with scrambled (non-targeting) siRNA (lane 1), a pool of four siRNa oligos directed against USP14 (lane 2), or individual oligos targeting USP14 (lane 3 and 4). Cells were lysed and incubated with probe **2** and DUBs were visualized by in-gel fluorescence. A decrease in USP14 reactivity with the probe in lane 2, 3 and 4 confirms the loss of functional USP14 in the cells transfected with siRNA targeting USP14.

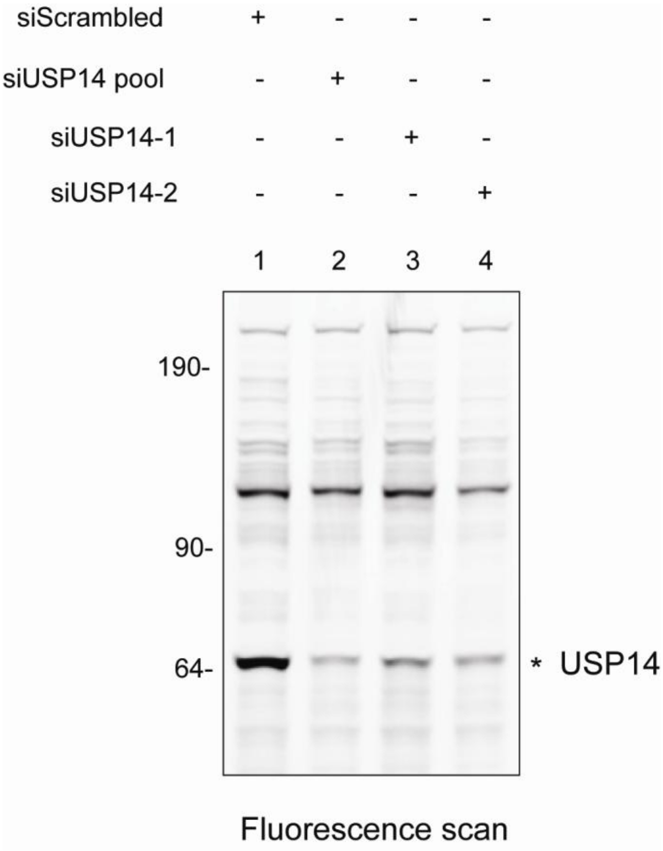

Figure S14. **Fluorescent DUB activity probe 2 allows for direct comparison of differential activity profiles.** Androgen receptor (AR) signalling is important in all stages of prostate cancer and androgen deprivation through chemical castration or use of anti-androgens are frequently used in therapy. We examined DUB activity in the LNCaP derivatives, LNCaP-AI (androgen independent) and LNCaP-CdxR that were continuously cultured in androgen depleted conditions or in the presence of the anti-androgen Casodex (CAS), respectively, that are representative of advanced prostate cancer. We also manipulated androgen levels in LNCaP cells using the androgen receptor agonist dihydrotesterone (DHT) or androgen antagonist Casodex to examine the response to AR transcriptional activation or inhibition. DUB activity profiles were examined by treating lysates with **2**. For the different conditions tested, no UCHL1 activity was observed. These data suggest UCHL1 activity is independent of AR status.

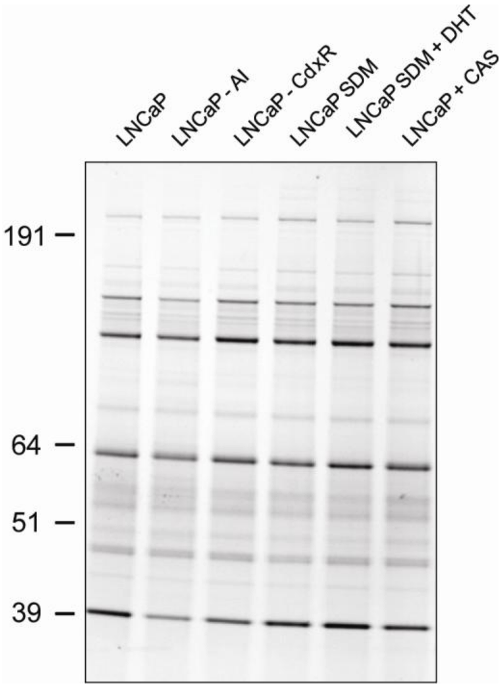

Experimental section Figure S14

LNCaP cells were grown in RPMI 1640 medium (Gibco) supplemented with 10% (v/v) fetal calf serum (FCS) and 2 mM L-glutamine at 37 ˚C in 5% CO_2 atmosphere. LNCaP-CdxR cells were generated and maintained continuously in 2 µM Casodex, as described previously.** LNCaP-AI cells were generated in house by serially maintaining LNCaP cells in steroid depleted media (SDM) for >9 months. For androgen manipulation experiments, LNCaP cells were grown in SDM for 72 hours and then transferred to SDM +/- 100nM dihydrotestosterone (LNCaP + DHT) for a further 24 hours. LNCaP cells were also treated with the androgen antagonist, Casodex (1 µM) for 24 hours (LNCaP + CAS) to block androgen receptor activity.

****** a) K. Halkidou, V. J. Gnanapragasam, P. B. Mehta, I. R. Logan, M. E. Brady, S. Cook, H. Y. Leung, D. E. Neal, C. N. Robson, *Oncogene* 2003, *22*, 2466-2477; b) A. C. Rigas, C. N. Robson, N. J. Curtin, *Oncogene* 2007, *26*, 7611-7619.

Figure S15. **The presence of labeled and unlabeled UCHL in DU145 cell extract was confirmed using UCHL1 antibody.** DU145 cell extract (2 mg/mL) was incubated with 1 μM probe **4** as described in the methods section. Following SDS-PAGE analysis, proteins were blotted onto PVDF membrane and stained with anti-UCH-L1 (Enzo Life Sciences) (dilution 1:2000) and Swine Anti-Rabbit HRP (Dako) (dilution 1:10000) as secondary antibody. Proteins were visualized as described in the methods section. UCHL1 was present in DU145 cell extracts as shown by anti-UCHL1 staining. The UCHL1 band migrated at the same molecular weight as the abundant fluorescent DUB present in DU145 cell extract.

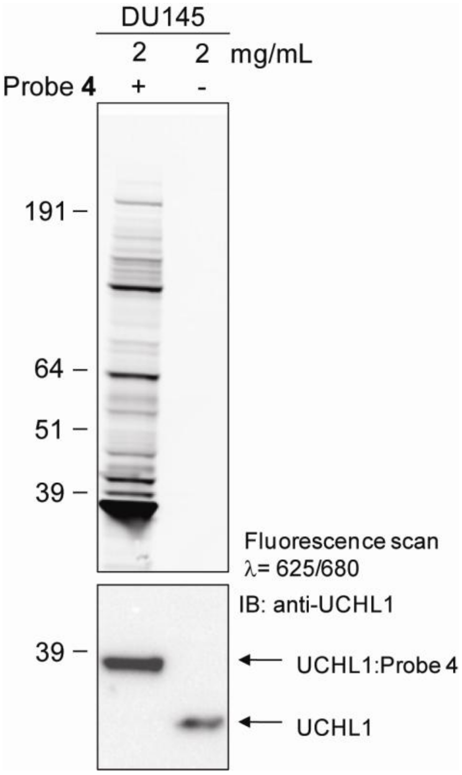

Supplement: Supplementary file 1 [file cbic0013-0293-SD1.pdf]
